# Transpedical Interbody Bone Grafting in the Treatment of Senile Osteoporotic Vertebral Fracture

**DOI:** 10.12669/pjms.335.12908

**Published:** 2017

**Authors:** Zhiwei Qin, Hong Liu, Guiying Chen, Guifeng Liu, Peng Zhang, Haitao Zhu

**Affiliations:** 1 Zhiwei Qin, Spine Surgery Department, Taian City Central Hospital, Shandong - 271000, China; 2 Hong Liu, Spine Surgery Department, Taian City Central Hospital, Shandong - 271000, China; 3 Guiying Chen, Obstetrical Department, Taian City Central Hospital, Shandong - 271000, China; 4 Guifeng Liu, Spine Surgery Department, Taian City Central Hospital, Shandong - 271000, China; 5 Peng Zhang, Spine Surgery Department, Taian City Central Hospital, Shandong - 271000, China; 6 Haitao Zhu, Spine Surgery Department, Taian City Central Hospital, Shandong - 271000, China

**Keywords:** Senile osteoporosis, Spinal fracture, Transpedical interbody bone grafting

## Abstract

**Objective:**

To evaluate the clinical effect of transpedical interbody bone grafting in the treatment of senile osteoporotic vertebral fracture.

**Methods:**

Eighty-six elders with osteoporotic vertebral fracture were selected and divided into a control group and a test group using random double-blind method. Patients in the control group were treated by short-segment transpedicular screw system internal fixation, while patients in the test group were treated by short-segment transpedicular screw system internal fixation in combination with transpedical interbody bone grafting. Operation related indexes and fracture recovery condition were compared between the two groups.

**Results:**

The overall effective rate of the test group was 93.02%, much higher than the control group (76.74%) (P<0.05). The difference of operation duration, intraoperative bleeding volume, length of hospital stay, fracture healing time, preoperative vertebral height loss and preoperative Cobb’s angle between the two groups had no statistical significance (P>0.05). The postoperative pain score of the test group was lower than that of the control group, and the difference was statistically significant (P<0.05). The vertebral height loss and Cobb’s angle of the test group were superior to those of the control group at the last follow up, and the difference had statistical significance (P<0.05). The incidence of internal fixator loosening of the test group was much lower than that of the control group (P<0.05).

**Conclusion:**

Short-segment transpedicular screw system internal fixation in combination with transpedical interbody bone grafting shows favorable effects in the treatment senile osteoporotic vertebral fracture, resulting in mild pain and less loss of vertebral height and angle; hence it is worth promotion in clinic.

## INTRODUCTION

Osteoporotic vertebral fracture is common among elders, which manifests as convex deformation of spinal cord and waist and back pain. Patients with osteoporotic vertebral fracture who has pure pain can be firstly treated by conservative treatment including drug treatment, stay in bed and wearing support. Those who have severe vertebral posterior process and have no response to conservative treatment can undergo surgical treatment.^1^,^2^ With the acceleration of aging in recent years, the incidence of osteoporotic vertebral fracture suggests a tendency of significant increase; hence the treatment of elder osteoporotic vertebral fracture has been extensively concerned.^3^ Senile osteoporotic vertebral fracture is mainly treated by short-segment transpedicular screw system internal fixation which is featured by simple operation, good stability, small trauma and good effect. However, as vertebral body is a kind of cancellous bone which is easy to crack and be compressed once being fractured, internal fixation system may be loosen and fractured due to the concentration of stress. Especially for elders with weakened physical function, the incidence of internal fixation failure is higher.^4^,^5^ In recent years, with the constant development of medical technology, transpedicular grafting on the basis of internal fixation has been attempted in clinic to narrow the gap after restoration, reconstruct spinal stability, and promote better healing.^6^

This study retrospectively analyzed 86 elders with osteoporotic vertebral fracture who were admitted into the Taian City Central Hospital, aiming to deeply investigate the clinical effects of transpedical interbody bone grafting.

## METHODS

### General data

Eighty-six elders with osteoporotic vertebral fracture who were admitted into the hospital from May 2014 to May 2015 were selected as research subjects and randomly divided into a test group and a control group. The test group (n=43) included 24 males and 19 females; they aged 60~75 years old (average 68.9±2.4 years old); the time from injury to operation was 43.14±10.25 h averagely; there were 13 cases of traffic accident induced injury, 18 cases of falling induced injury, and 12 cases of bruises; the fracture segments included T_11_ (4 cases), T_12_ (9 cases), L_1_ (7 cases), L_2_ (12 cases), and L_3_ (11 cases). In the control group (n=43), there were 25 males and 18 females; they aged 61~74 years old (average 68.9±1.5 years old); the time from injury to operation was 43.11±10.18 h averagely; there were 16 cases of traffic accident induced injury, 15 cases of falling induced injury, and 12 cases of bruises; the fracture segments also included T_11_ (5 cases), T_12_ (8 cases), L_1_ (6 cases), L_2_ (13 cases), and L_3_ (11 cases). The comparison of general data between the two groups suggested no significant difference (P>0.05); therefore, the results were comparable. This study has been reviewed and approved by the ethics committee of the hospital. All patients signed informed consent before study.

### Inclusive criteria

Patients who would undergo short-segment transpedicular screw system internal fixation, had fracture in T_11_~L_3_, were treated by the same group of doctors, and were followed up for more than 6 months were included. But those who have undergone surgery through anterior and posterior approach or anterior approach, had paraplegia, or underwent vertebral posterolateral bone grafting were excluded.

### Treatment methods

Patients in the two groups took prone position after being treated by general anesthesia. According to the imaging examination results, the fracture plane was taken as the center and an incision was cut around facet joint, cone plate and spinous process through posterior midline approach. Patients in the control group underwent short-segment transpedicular screw system internal fixation only. The operative approach was the V-shape ridge of unilateral or lateral articular process of fracture vertebra body and the injured vertebra was installed with connecting rods and pedicle screws. After that, the patients were re-examined using C-arm X-ray machine. If the restoration effect was poor, part of vertebral plate needed to be cut. Moreover, the bone intruding to spinal canals were knocked to promote fracture reduction. Besides short-segment transpedicular screw system internal fixation, patients in the test group were inserted with needles at unilateral or bilateral pedicle of vertebral arch and installed with pedicle screws. If there was no rupture, artificial bone was implanted using a push rod and the entrance was smeared with bone wax. After operation, patients in the two groups were given antibiotics for preventing infection.

### Observation indexes and criteria of curative effect

The determination criteria of clinical effects were as follows.^7^ Treatment was determined as ineffective if fracture symptoms such as pain and movement disturbance were not relieved, imaging examination suggested fracture was not healed, and there was obvious abnormality in spinal function. Treatment was considered as effective if fracture symptoms such as pain and movement disturbance were significantly relieved, imaging examination suggested fracture was almost healed, and the spinal functions were obviously relieved. Treatment was determined as significantly effective if all fracture symptoms such as pain and movement disturbance thoroughly disappeared, imaging examination suggested complete healing of fracture, and spinal function recovered to normal level or the previous level. Overall effective rate was calculated using the following formula: overall effective rate=(number of cases obtaining significant effect+number of cases obtaining effective effect)/total number of cases×100%. Clinical indexes including duration of operation, intraoperative bleeding volume, fracture healing time, length of hospital stay and postoperative pain score were recorded. Pain score was determined using visual analogue scale (VAS); 0 point stands for no pain and 10 points stands for unbearable pain. Values of indexes which could reflect fracture recovery conditions such as preoperative vertebral height compression, postoperative Cobb’s angle, postoperative vertebral height loss, vertebral height loss at the last follow up and Cobb’s angle at the last follow up as well as the incidence of internal fixation loosening were recorded.

### Statistical analysis

Data were processed by SPSS ver. 21.0. Enumeration data were expressed by percentage and the comparison between groups adopted X^2^ test. Measurement data were expressed by mean±SD and the comparison between groups was performed using t test. Difference was determined as statistically significant if P<0.05.

## RESULTS

### Clinical effects

**Fig.1:**
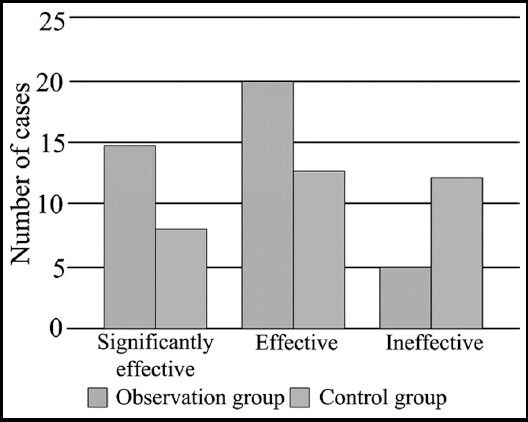
Comparison of treatment effects between the two groups.

**Fig.2:**
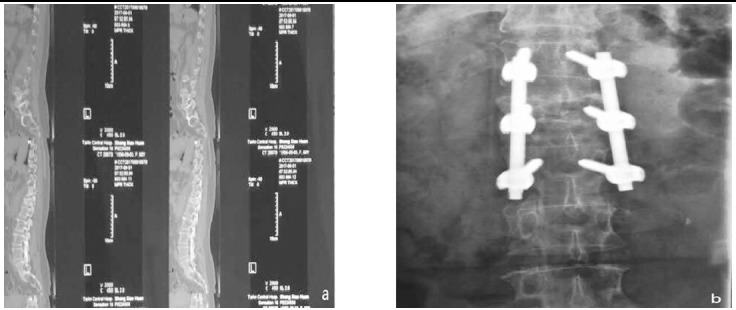
Female, aged 61 years old, a: T-12 vertebral compression fracture before surgery (IV degree); b: screw-rod internal fixation after surgery and vertebral body graft.

**Fig.3:**
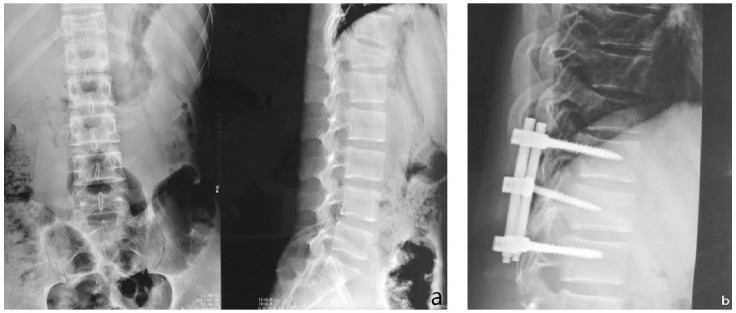
Male, aged 57 years old, a: T-12 vertebral compression fracture before surgery (III degree); b: screw-rod internal fixation after surgery and and no graft for the diseased vertebra.

The overall effective rate of the test group was much higher than that of the control group (93.02% vs 76.74%); the difference had statistical significance (X^2^=5.92, P<0.05) (Fig.1). The surgical conditions of the typical cases in the two groups are shown in Fig. 2 and 3.

### Comparison of operation related clinical indexes between the two groups

The comparison of duration of operation, intraoperative bleeding volume, length of hospital stay and fracture healing time between the two groups suggested no statistically significant difference (P>0.05) (Table-I).

**Table-I:** Comparison of operation related clinical indexes between the two groups (Mean±SD).

*Group*	*Test group*	*Control group*	*t*	*P*
Duration of operation (min)	180.4±48.9	179.7±66.4	1.513	>0.05
Intraoperative bleeding volume (mL)	390.5±90.3	384.6±89.5	0.023	>0.05
Length of hospital stay (d)	22.0±4.5	23.1±5.6	0.804	>0.05
VAS score (point)	1.76±1.09	5.22±2.90	8.196	<0.05
Fracture healing time (week)	6.34±1.12	6.25±1.17	0.082	>0.05

### Comparison of fracture recovery conditions between the two groups

The comparison of preoperative vertebral height, postoperative vertebral height and postoperative Cobb angle between the two groups suggested no remarkable difference (P>0.05). But the vertebral height loss and Cobb’s angle of the test group was superior to those of the control group at the last follow up, and difference had statistical significance (P<0.05) (Table-II).

**Table-II:** Comparison of fracture recovery condition between the two groups.

*Group*	*Test group*	*Control group*	*t*	*P*
Preoperative vertebral height compression (%)	46.7±14.5	47.6±16.8	0.542	>0.05
Postoperative vertebral height loss (%)	4.08±4.02	6.17±5.19	0.303	>0.05
Vertebral height loss at the last follow up (%)	4.71±3.24	10.99±3.27	5.064	<0.05
Postoperative Cobb’s angle	24.36±7.37	24.92±7.96	0.592	>0.05
Cobb’s angle at the last follow up (°)	5.03±3.24	11.22±6.27	4.472	<0.05

### The comparison of the incidence of internal fixation loosening

The patients were followed up for 6 to 12 months. One patient in the test group (2.33%) and seven patients in the control group (16.28%) were found with internal fixation loosening, and the difference between the two groups was statistically significant (X^2^=9.239, P<0. 05).

## DISCUSSION

The follow up of elders who have osteoporotic vertebral fracture and underwent internal fixation suggested that, many patients had wedging changes in fracture vertebra body. It is because some cancellous bones at collapse position cannot be successfully opened during surgery though local injured site has been processed by distraction and the shape of vertebrae has been restored to some extent, and as a result some bones in certain range are in an empty state and carry excessive gravity. Especially for elders, the gravity is difficult to be tolerated and the risks of wedging changes in fracture vertebra body, leading edge lowering and kyphos are significantly higher after the removal of internal fixation, which can severely affect the long-term efficacy of surgery.^8^,^9^ It has been pointed out that; short-segment transpedicular screw system internal fixation can inevitably lose treatment effect with the increase of weight-bearing activities, though it can achieve favorable restoration effect in the initial stage.^10^ A recent study applied external implantation of artificial bone after short-segment transpedicular screw system internal fixation to prevent malformation and the loss of corrective angle and height and achieved certain improvement effect,^11^ but the treatment effect was not satisfactory.

In this study, the incision of transpedical interbody bone grafting was consistent with the incision of internal fixation and the surgery was featured by simple operation and small trauma. Moreover, the surgery could effectively avoid the concentration of stress at the position of transpedicular screw and reduce the probability of loosening and fracture; vertebral superior endplate and inferior endplate were supported by the implanted bone, which could stabilize fracture and also promote the healing of fracture.^12^-^16^

In the study, the pain score of the test group was lower than that of the control group after internal fixation (P<0.05), which might be because of the instable fractured vertebrae and cavity. But the implantation of artificial bone improved the stability and relieved pain. The incidence of vertebral height loss, Cobb’s angle and internal fixator failure of the test group was superior to that of the control group at the last follow up (P<0.05), suggesting the superiority of transpedical interbody bone grafting in the treatment of senile osteoporotic vertebral fracture.

### Limitations of the study

During study, few complications occurred to the patients. The research results were simple because of the small size of samples and limited research time and grant. The prognosis condition such as the improvement of living quality was not deeply investigated. Studies with large sample size can be carried out in the future.

## CONCLUSION

In conclusion, transpedical interbody bone grafting is of great help to the treatment of senile spinal fracture as it can improve the stability after surgery, relieve pain, reduce the loosening and fracture of internal fixator and the loss of vertebral corrective angle and height, improve surgical efficacy, and increase the satisfaction of patients. Therefore, it is worth promotion in clinics.

### Authors’ Contribution

**ZWQ, HL & GYC:** Study design, data collection and analysis.

**GFL, PZ & HTZ:** Manuscript preparation, drafting and revising.

**ZWQ & HL:** Review and final approval of manuscript.
